# Observations of feeding practices of US parents of young children with Down syndrome

**DOI:** 10.1111/mcn.13548

**Published:** 2023-07-17

**Authors:** Victoria A. Surette, Sarah Smith‐Simpson, Lisa R. Fries, Ciarán G. Forde, Carolyn F. Ross

**Affiliations:** ^1^ School of Food Science Washington State University Pullman Washington USA; ^2^ Sensory and Consumer Insights Nestlé Nutrition North America (Gerber) Fremont Michigan USA; ^3^ Nestlé Research Beijing China; ^4^ Department of Agrotechnology and Food Sciences Wageningen University & Research Wageningen the Netherlands

**Keywords:** behavioural observation, children, Down syndrome, eating behaviour, parental feeding practices, trisomy 21

## Abstract

Parental behaviours influence food acceptance in young children, but few studies have measured these behaviours using observational methods, especially among children with Down syndrome (CWDS). The overall goal of this study was to understand parent feeding practices used during snack time with young CWDS (*N* = 111, aged 11–58 months). A coding scheme was developed to focus on feeding practices used by parents of CWDS from a structured home‐use test involving tasting variously textured snack products. Behavioural coding was used to categorise parental feeding practices and quantify their frequencies (*N* = 212 video feeding sessions). A feeding prompt was coded as successful if the child ate the target food product or completed the prompt within 20 s of the prompt being given without a refusal behaviour. CWDS more frequently consumed the test foods and completed tasks in response to Autonomy‐Supportive Prompts to Eat (49.3%), than to Coercive‐Controlling Prompts to Eat (24.2%). By exploring the parent–CWDS relationship during feeding, we can identify potentially desirable parent practices to encourage successful feeding for CWDS. Future research should build upon the knowledge gained from this study to confirm longitudinal associations of parent practices with child behaviours during feeding.

## Key messages

- A coding scheme was developed to focus on feeding practices used by parents of children with Down syndrome (CWDS).
- Parents of CWDS were observed to use more autonomy‐supportive feeding practices to convince their children to eat the target food compared to coercive‐controlling practices.
- Parents and feeding practitioners for CWDS may consider using supportive feeding practices to encourage acceptance in this population.

## 1 INTRODUCTION

Parent–child interaction during feeding is important, as it contributes to the development of children's eating behaviour and food preferences (Polfuss et al., 2017; Savage et al., 2007). Parents influence their children's feeding by providing food and creating a feeding environment during consumption, which in turn can impact the child's early experiences with food (Kral & Rauh, 2010; Savage et al., 2007). Encouraging healthy eating habits may be the intent of a parent during mealtime, but specific behaviours used by parents during feeding, such as controlling practices, may have a negative impact on child eating habits, eating behaviour, body composition and nutrient and energy intakes (Blissett, 2011; Fogel et al., 2019; Fries & van der Horst, 2019; Fries et al., 2017; Savage et al., 2007; Shloim et al., 2015; Wehrly et al., 2014). By contrast, evidence suggests that modelling healthy eating behaviours can improve a child's food acceptance (Fries & van der Horst, 2019; Palfreyman et al., 2015). Parent‐feeding practices during child feeding have been extensively studied in children with typical development (CTD), but much less research has been conducted among populations with developmental and/or intellectual delays, such as children with Down syndrome (CWDS; Polfuss et al., 2017). DS is the most common chromosomal condition in the United States, with approximately 1 in every 707 babies born diagnosed with DS (CDC, 2020; Mai et al., 2019). Feeding challenges, and specifically texture selectivity, are more prevalent in CWDS compared to CTD (van Dijk & Lipke‐Steenbeek, 2018; Ross et al., 2021). Approximately 80% of CWDS have oral motor delays that may contribute to these challenges (Field et al., 2003). Understanding the impact these challenges impose on child food acceptance and the resultant parental feeding practices is important for the development of evidence‐based guidelines to support parents aiming to promote healthy diets.

In addition to food rejection, another challenge that may influence the parental feeding practices used by parents of CWDS is the concern for childhood obesity among this population. O'Neill et al. (2005) compared parental feeding behaviours between CWDS and their siblings (*n* = 36, aged 3–10 years) and reported parents tended to use controlling behaviours more frequently for CWDS than for their siblings during feeding. This was attributed to the parents' increased concern about obesity in their CWDS, prompting increased use of controlling feeding behaviours (Costanzo & Woody, 1985). Since the obesity risk in CWDS has been observed to increase after age 2 (Bertapelli et al., 2016), parent feeding behaviours may already be affected during the toddler years (Ek et al., 2016; Polfuss & Frenn, 2012; Polfuss et al., 2017).

The increased use of controlling feeding practices was not observed in general parenting practices outside of a feeding context (Phillips et al., 2017). This observational result in general parenting of CWDS is interesting in comparison to the parent report of increased use of controlling feeding behaviours during feeding of CWDS from O'Neill et al. (2005). Further research is needed to determine if there is a parent behaviour change from general parenting to feeding CWDS. Accompanying parent‐report surveys with observational methods in both general parenting and feeding contexts of CWDS will answer this question.

Of the few studies that have explored parenting and CWDS (*n* = 35, CWDS aged 5–9 years, Phillips et al., 2017) and parenting dimensions in CWDS (n = 25, CWDS aged 4–6 years, Gilmore & Cuskelly, 2012; *n* = 10, CWDS aged 30–39 months, Blacher et al., 2013), researchers have utilised parent self‐report surveys without direct observation of parent or child behaviour. The inclusion of observational measurements alongside parent report questionnaires in exploring parent–child mealtime interactions have been used in several published feeding studies in CTD (e.g., Edelson et al., 2016; Fogel et al., 2019; Fries et al., 2017; Fries, Chan, et al., 2019; Jordan et al., 2020; Orrell‐Valente et al., 2007; Palfreyman et al., 2015; Pesch & Lumeng, 2017; Power et al., 2021). No studies to date have combined both observational measures with parent reports within CWDS dyads during feeding. Utilising both methodologies can aid in the comparison of the same response from two different perspectives, for instance, if researchers are observing what parents are observing/reporting. Additionally, the utilisation of both methods can provide a large context of the feeding experience from parent perception to the objective interpretation of parent feeding practices.

Exploring the relationship between parent feeding practices and CWDS feeding responses is important to inform the development of guidance on the most effective practices used by parents that facilitate a positive feeding experience (e.g., decreased stress, increased food exploration; Caldwell & Krause, 2021). The overall goal of this study was to understand parent feeding practices used during snack time with young CWDS. For the observational measures, video data from a home‐use test was used to explore the parent–CWDS relationship during feeding (Surette et al., 2021). The home‐use test evaluated child eating behaviours and acceptance of commercial solid snack food products of various textures in a CWDS population. A behavioural coding scheme was developed to quantify parental feeding practices during snack time. We hypothesised that parents of CWDS would use more supportive feeding practices (e.g., Autonomy‐Supportive Prompts to Eat) than controlling feeding practices (e.g., Coercive‐Controlling Prompts to Eat) during the feeding sessions, based upon the observational results from the Phillips et al. (2017) that reported no observations of increased use of controlling feeding practices in general parenting practices. We further hypothesised that parents would be consistent in their behaviours/practices across two observed days.

## 2 MATERIALS AND METHODS

### 2.1 Home‐use test overview

A home‐use test was developed to compare food texture acceptance and identify mealtime behaviours in CWDS. A detailed description of the methods, participant eligibility criteria and textured products is provided by Surette et al. (2021) and the results have been published by Ross et al. (2022).

In summary, the test involved shipping four products and additional study materials (e.g., video recording and feeding study instructions) to the homes of participants. The study was completed over six consecutive days and participants were asked to evaluate each test food once per day. Parents recorded their own liking of the food products as well as their perception of their child's liking of the food products using a nine‐point scale presented through an online platform. Parents were instructed to provide their children with their normal feeding environment, use a consistent location for filming every day, and to minimise distractions and facial gestures that might influence their child's evaluation of the food. From the videos, a panel of trained coders used a pre‐defined behavioural coding scheme to capture parents' verbal and non‐verbal behaviours during snack time, as well as child mealtime behaviours and food acceptance.

To reduce the number of videos that needed to be coded to explore parent feeding practices in response to a new task and the same feeding practices after the parent and child were used to the task, 2 of the 6 days of video recordings were selected as a representative for behavioural annotation and coding. To determine which of the 6 days to include, several analyses of variance (ANOVA) were conducted. Results from the home‐use test showed that the overall disposition of the children to foods on early feeding days (Days 1–3) did not significantly differ (*p* > 0.05); no significant differences were noted on later feeding days (Days 4, 5 and 6 either. Thus, Day 2 was selected as it was in the middle of the early study days, while Day 5 was in the middle of the later study feeding days.

### 2.2 Participants and recruitment

A total of *N* = 111 parent–CWDS (11–58 months of age) dyads were included in the home‐use test and participant demographics are presented in Table 1. The study population was nationally recruited in the United States between April 2016 and March 2018. Recruitment consisted of contacting DS organisations, social media outreach, and recruitment by the study team at the National Down Syndrome Congress Convention in July 2017 (Sacramento, CA). Recruited participants consented to complete a screening questionnaire to determine study qualification. Compensation was provided for the CWDS participants who qualified and completed the study (*N* = 111).

**Table 1:** Participant characteristics (*N* = 111).

Characteristic	Value
Parental age in years (mean, SD)	36.3 (4.9)
Parental gender (*n*, %)	
Female	107 (96.4)
Male	4 (3.6)
Parental marital status (*n*, %)	
Divorced/separated	0 (0)
Living with partner	2 (1.8)
Married	107 (96.4)
Single (never married)	2 (1.8)
Widowed	0 (0)
Parental race (*n*, %)	
American Indian/Native Alaskan	1 (0.9)
Asian	4 (3.6)
Black/African American	2 (1.8)
Native Hawaiian/Pacific Islander	1 (0.9)
White	105 (94.6)
Child age in months (mean, SD)	31.4 (13.2)
Child sex (*n*, %)	
Female	44 (39.6)
Male	67 (60.4)
Child race (*n*, %)	
American Indian/Native Alaskan	0 (0)
Asian	6 (5.4)
Black/African American	3 (2.7)
Native Hawaiian/Pacific Islander	1 (0.9)
White	102 (91.9)
Other	4 (3.6)
Household income (mean)	$80,000–89,999
Household setting (*n*, %)	
Urban (city centre)	13 (11.7)
Suburban (city surroundings)	75 (67.6)
Rural (country setting)	23 (20.7)
Total state participation (*n*)	35
USA region representation (*n*, %)	
Northeast	15 (13.5)
Midwest	28 (25.2)
South	35 (31.5)
West	33 (29.8)

### 2.3 Coding scheme development

The coding scheme used was developed to focus on the feeding techniques used by parents with CWDS. The coding scheme incorporated elements from previously established coding schemes of feeding practices (Edelson et al., 2016; Fries et al., 2017; Fries, van der Horst, et al., 2019; Orrell‐Valente et al., 2007; Surette et al., 2021) and was applied to each child's feeding session. Qualtrics software (Version April–August 2021) was used to collect all data pertaining to the coding scheme of the parent feeding behaviours.

#### 2.3.1 Coding periods

For each feeding session, parental feeding practices and child eating behaviours were coded across the three distinct periods of the eating occasion: (i) baseline state, (ii) initial presentation and (iii) food engagement as were used in our previous study (Surette et al., 2021). The baseline state included the period during which the child was waiting for a product to be presented to them. The initial presentation was the period between the child looking at the product and the child trying/rejecting the product. Food engagement was defined as the period between the child trying/rejecting the product, and the child finishing the product or the parent taking the product away from the child.

#### 2.3.2 Preliminary coding scheme

Before the official coding of the CWDS video data, the lead researcher/coder designed and performed a preliminary study with the first version of the coding scheme. The preliminary coding scheme consisted of eight feeding practices which have previously been defined by Fries et al. (2017) and Fries, van der Horst, et al. (2019): Autonomy‐Supportive Prompts to Eat (ASP), Coercive‐Controlling Prompts to Eat (CCP), Hurrying, Slowing, ‘Other’, Positive Talking, Negative Talking, Neutral Talking. Eighteen CWDS videos were randomly selected and coded, with a balance of the day (1–6 of the home‐use test feeding study) and sex (male or female). The coded behaviours are summarised in Table 2.

**Table 2:** Coding scheme and corresponding examples of feeding practices per video period.

Video period	Coding	Feeding practice	Example
Baseline State: *Period in which the child was waiting for a product to be presented to them.* Initial Presentation: *Period between the child looking at the product and the child either trying/rejecting the product.* Food Engagement: *Period between the child trying/rejecting the product, and the child finishing the product or the parent taking the product away from the child.*	Success^a^ (2‐pt scale)	**Autonomy‐Supportive Prompts to Eat**	− Mom eats the product and says, ‘Yum!’ − ‘Try your hot dog’, in a neutral/positive tone. − ‘You want to take another bite?’
		Defined as giving a prompt to the child in a gentle, non‐coercive way. The child maintains the ability to decide whether to eat/complete the prompt or not.	
		**Coercive‐Controlling Prompts to Eat**	− ‘If you eat three more bites of meat, you can have a popsicle’. − ‘Eat it, don't waste food’. − ‘If you won't eat, I'm taking away the iPad’.
		Defined as giving a prompt to the child in a coercive manner, either through pressure or bribery.	
		**Instruction**	− ‘Use your spoon’. − ‘Chew it up’. − ‘Be careful, it's hot’.
		Defined as an instruction on how to perform an action that the child is doing.	
		**Interference**	− ‘Stop that!’ − ‘Hold on!’ in a harsh/negative tone. − ‘No!’ in a harsh/negative tone.
		Defined as an attempt to change or restrain the child's behaviour.	
		**Water**	− ‘Want some water?’
		Defined as asking the child to take a drink.	
		**Other**	− Playing a game with the food. − Singing a song. − Asking the child to say something unrelated to the product.
		Defined as any prompts not directly included in the coding scheme or involving the food product.	
	Count	**Hurrying** b	− ‘Hurry up, eat your honey stars’. − ‘Faster’.
		Defined as encouraging the child to eat faster or within a certain amount of time.	
		**Slowing** b	− ‘Slow down’. − ‘Take your time’.
		Defined as asking the child to eat more slowly.	
		**Positive Talking** c	− ‘Are you ready for your next snack?’ − ‘You're a good girl, you're eating by yourself’. − ‘You ate all your chicken, good job!’
		Defined as making a positive comment about the child's food, using a positive tone of voice, or showing approval of child's eating.	
		**Negative Talking** c	− ‘I don't like this’. − ‘Eat properly, why do you always eat like that?’
		Defined as making a negative comment about the child's food or eating and using a negative tone of voice.	
		**Neutral Talking** c	− Asking questions about the food. − ‘Here you go’. − ‘Are you all done?’ in a neutral tone.
		Defined as making a comment that is not obviously positive or negative.	
Overall feeding session	4‐pt scale	**Amount of Sample Consumed**	**Coder responses:**
			All = 3
			More than half (not all) = 2
		Defined as the amount of product consumed by the child.	Half or less = 1
			None = 0
	5‐pt scale	**Overall Disposition** d	**Coder responses:**
			Strongly negative = −2
			Negative = −1
		Defined as the child's overall disposition to the product.	Neutral = 0
			Positive = 1
			Strongly positive = 2

^a^ A successful child response means that the child ate and/or followed the prompt direction without a refusal within 20 s of the prompt.

^b^ Counts were made for these feeding practices in case they were present in the data set, as none were observed in the preliminary data set.

^c^ Counts were made for these feeding practices, as the talking practices are not indicative of giving a child a specific prompt/task.

^d^ This response scale was consolidated to a 3‐point scale for all analyses, with strongly negative and negative combining into negative (−1), with neutral remaining the same (0), and with strong positive and positive combining into (1).

#### 2.3.3 Final coding scheme

The final coding scheme was modified with respect to the type and amount of feeding practices incorporated. Since no ‘Hurrying’ or ‘Slowing’ were observed during the preliminary study, these practices were converted from being coded as prompts to then being counted as distinct point events (not coded as a prompt). These two feeding practices were kept in the coding scheme to capture the practices if present during the final coding of the CWDS data set so as to not miss these behaviours that other feeding studies have reported.

Another modification of the preliminary coding scheme was the addition of the ‘Interference’, ‘Instruction’ and ‘Water prompt’ categories. It was necessary to further distinguish the CCP from Interference parent practices, and ASPs from the Instruction parent practices. This allowed coders to account for differences in parental practices with respect to both the food product and the parent practice that either improved a child's action (Instruction practice) or discouraged certain child behaviours during snack time (Interference practice). The ‘Water prompt’ category was introduced to distinguish between prompts to drink from the other prompts to eat.

Table 2 lists the feeding practice, its definition, how the practice was coded, and corresponding examples encountered by previous observational studies and the current study. It also includes how the amount of food consumed and the child's overall disposition to the foods were coded. Autonomy‐Supportive Prompts to Eat (ASP), Coercive‐Controlling Prompts to Eat (CCP), Instruction prompts, Interference prompts, Water prompts and ‘Other’ prompts were feeding practices coded as successful or unsuccessful. Hurrying, Slowing, Positive Talking, Negative Talking and Neutral Talking were feeding practices coded as a count. Coders could indicate comments at the end of each video period to provide additional details about the practice or context.

A prompt was coded as successful if the child ate the target food or completed the prompt within 20 s of the prompt being given without a refusal behaviour (Edelson et al., 2016). A refusal behaviour was defined as the child turning their head away, increasing distance from the stimulus, throwing food, verbally saying ‘no’, or similar (Surette et al., 2021). Unsuccessful prompts were those that had a refusal behaviour or did not complete the prompt/eat the target food within 20 s of the prompt being given. An example of a feeding practice that is nonfood related is a parent asking their child to, ‘Say “hi” to the camera’, and a successful coded child response would be the child saying ‘hi’ to the camera. Prompts were also coded as unsuccessful if the child did not have the ability to complete the prompt (e.g., parent prompted the child to eat the food, but there was no food in front of the child within the 20 s timeframe). If multiple prompts (same or different type) were given within the 20 s allotment, those prompts were not counted if the child had not responded to the first prompt.

### 2.4 Coder training

Coders were trained using methods similar to those described by Surette et al. (2021) and Edelson et al. (2016). Coders received training materials before training that included: the project overview, the coding scheme, feeding practice definitions and examples, video period timings (i.e., baseline state, initial presentation and food engagement periods), and serving orders per child participant.

Training (2 h/day) occurred over three consecutive days with six randomly selected videos from the data set. The lead researcher reviewed the coding of feeding practices and led the practice coding of two videos on the first day. On the second and third days of training, the lead researcher led the coding of one video and then allowed for separate coding of another video before discussion.

After the initial 6 h of training, the lead researcher and two coders each coded 24 videos randomly selected by the lead researcher. Per cent agreement was the measurement used to monitor the reliability between the two coders for all videos in the study since the presence of zeros is often an issue with behavioural coding. Once the goal of >80% agreement was reached, the coding of the study videos began (Edelson et al., 2016; Surette et al., 2021). The statistical analysis of inter‐coder reliability was performed using Stata v.14 (Stata Corporation). All videos, 222 videos (Days 2 and 5 per child participant), were reviewed by at least two coders. All questions about coding, including disagreements and clarifications, were resolved by the lead researcher.

The training video data set results from the reliability analysis indicated that >80% agreement would be very difficult to achieve with the subjectivity of the ASP, Positive Talking and Neutral Talking practices. Therefore, a reliability analysis and count measurement were conducted at the end of each week of the official coding (10 weeks with 20 videos coded per week per coder, and 1 week with 22 videos coded per coder). The count measurement consisted of the lead researcher counting the number of ASP, Positive Talking and Neutral Talking practices, and ensuring the sum was the same per food product for each video coded. If coders failed to reach 80% agreement on their responses or the count measurement sum differed between coders, videos were re‐coded until sufficient agreement was met.

### 2.5 Statistical analysis

XLSTAT 16.0 (Addinsoft) was used to perform a paired *t*‐test to determine if the video lengths were the same across both days of the feeding study.

XLSTAT was used to perform paired t‐tests to determine whether parents were giving the same type and number of feeding practices to their child across both days, and to test the consistency of the feeding prompt success rates across both days. A two‐proportion *z*‐test was performed to determine if the success of prompts differed by type of prompt.

XLSTAT was used to perform an ANOVA to determine if specific feeding practices were used more for parents who fed their children during the home‐use test study compared to parents with children who fed themselves independently.

The distribution, mean number, and standard deviation of the feeding practices were calculated using Microsoft Excel (2021). The frequency of prompting the child to perform a task (i.e., number of ASP, CCP, Instruction, Interference, Water, and ‘Other’) per minute and the frequency of talking to the child (i.e., Hurrying, Slowing, Positive Talking, Negative Talking and Neutral Talking) per minute were also calculated using Microsoft Excel. Hurrying was removed from the analysis because there were no recorded counts of this feeding practice.

Content analysis of coder comments was performed in Microsoft Excel to explore additional behaviours occurring during the feeding sessions. Comments from every coded feeding session (Days 2 and 5) were categorised and counted.

### 2.6 Ethical statement

The home‐use test was approved by the Institutional Review Board of Washington State University (IRB #14706), with written informed consent obtained from all study participants.

## 3 RESULTS

The average video length coded was 11.5 min (±5.5 min). Days 2 and 5 video lengths were not significantly different from each other (*p* = 0.090).

Table 3 shows the mean number of feeding practices used overall, as well as the percentage of success of prompts overall. Overall, the average number of feeding practices experienced over one feeding session was approximately 21 prompts and 65 counts of talking. The frequency of prompting the child to perform a task per minute during a feeding session was 1.8 prompts per minute, while the frequency of talking to the child was 5.7 per minute. The most common feeding practices observed were positive talking (mean 8.1 times per video), autonomy‐supportive prompts (5.9), and neutral talking (4.0).

**Table 3:** Mean number of overall feeding practices (with standard deviation, SD), overall per cent success of feeding prompts and per cent success of feeding prompts for CWDS.

Feeding practice	Overall mean (SD)	Overall success of prompts (%)
Autonomy‐Supportive Prompts to Eat	5.9 (7.3)	49.3 c
Coercive‐Controlling Prompts to Eat	0.4 (1.6)	24.2 d
Instruction	1.4 (3.1)	69.3 a
Interference	0.6 (1.5)	46.1 c
Water	1.6 (2.7)	63.0 ab
Other	0.4 (1.6)	52.1 bc
Hurrying	–	–
Slowing	0.1 (0.4)	–
Positive Talking	8.1 (9.7)	–
Negative Talking	0.8 (2.7)	–
Neutral Talking	4.0 (20.1)	–

*Note*: Different letters within a column indicate significant differences at *p* < 0.05.

Abbreviation: CWDS, children with Down syndrome.

Overall, parents used more feeding practices on Day 2 than on Day 5 (*p* = 0.015). Specifically, more Neutral Talking was observed on Day 2 than on Day 5 (*p* = 0.003). Parents who fed their children used significantly more ASP, Instruction and Positive Talking, and more CCP than children feeding themselves independently (*p* < 0.05).

The overall success of the feeding prompts was not significantly different across both days of the feeding study (*p* = 0.230). The overall success of the feeding prompts across both days ranged from 24.2% (with CCP) to 69.3% (with Instruction). The success of feeding prompts depended on the type of prompt the parent used. CWDS successfully completed more prompts in response to ASP than CCP (*p* < 0.05).

Examples of ‘Other’ prompts that were not food‐related (*n* = 37 prompts as indicated by coder comments) included a parent asking the child to look at the camera and talk, asking the child to say a specific word or phrase, asking for the food product cup to be returned to the parent, and asking the child to sign for a specific item or action. Examples of ‘Other’ prompts that were food‐related (*n* = 23 prompts as indicated by coder comments) included the parent encouraging the child to play a game with the food, or the parent singing a song to get the child to eat the food.

Table 4 shows the results from the content analysis of the coder's comments from Days 2 and 5 during video coding. Seven content themes were observed from the comments: modelling (e.g., parent modelled eating); sign language (e.g., the parent used sign language to communicate); deviations from home‐use test directions (e.g., parent offered milk instead of water); obstacles for the child (e.g., the child fell asleep); distractions (e.g., siblings were distracting child); environment‐related prompts (e.g., the parent asked the child to say something) and positive experiences (e.g., the parent hugged the child).

**Table 4:** Content analysis of the number of overall (Days 2 and 5) coder comments from the baseline state, initial presentation and food engagement video periods. Examples of comments are indented under respective themes.

Comment theme	Video period		
	Baseline state	Initial presentation	Food engagement
Modelling	5	46	123
Parent modelled eating			
Parent modelled drinking			
Sign language	3	1	5
Parent used sign language to communicate			
Child signed that they were all done			
Deviations from home‐use test directions	6	0	19
Parent offered milk and/or juice instead of water			
Parent gave child too much product			
Parent gave child incorrect serving order			
Poor audio and video quality			
Obstacles for child	1	0	16
Child fell asleep or was sleepy			
Crumbs fell as child ate			
Child wanted powder from product off hands			
Child spit up food			
Child struggled to eat			
Distractions	0	0	28
Siblings were distracting child			
Neutral talking count was attributed to background talking			
Negative talking count was attributed to parents arguing			
TV or music was playing in the background loudly			
Environment related prompts	7	2	20
Parent asked child to say something			
Parent asked child to look at something (food and non‐food related) or someone			
Parent asked child to give the water cup or product cup back to the parent			
Positive experiences	0	7	30
Parent hugged child			
Parent gave child a toy to practice feeding on the toy			
Parent played a game or a song to encourage the child to eat			
Child played with food			
Child asked for more food			

The majority of comments from all themes were recorded while the child was in the food engagement video period. The most frequently reported comment theme was modelling within the food engagement period, with 123 counts of parents either modelling eating or modelling drinking water. During the initial presentation video period, the most frequently reported comment theme was modelling as well, with 46 counts. During the baseline state video period, the most frequently reported comment theme was environment‐related prompts (e.g., the parent asked the child to say something, and the parent asked the child to give the sample cup or water cup back to the parent).

## 4 DISCUSSION

The current study sought to observe parent feeding practices used during snack time with young CWDS when exposed to solid snack food products of various textures. Autonomy‐supportive prompts were more likely to convince CWDS to eat the target food (49.3% successful) than were controlling prompts (24.2%). Support through positive experiences included parents letting their children play with the food, parents hugging their children and parents encouraging their children to practice feeding on their toys. Since parents of CWDS used more ASP than CCP, and ASP was the more successful approach, this might suggest that parents have noticed that using ASP may be a more effective method to guide children's eating behaviour.

Since parents of CWDS may have more interactions with feeding specialists, this may be why they are utilising supportive practices when feeding CWDS (Marshall et al., 2015; Ross et al., 2019). Parents of CWDS have been encouraged to be attentive, use verbal encouragement, and teach new skills through play (Bruni, 2006). A previous study in CWDS during playtime also found that parental support elicited more engagement in play (Daunhauer et al., 2017).

Parental modelling of feeding behaviours has been observed to be an effective way to influence toddlers to eat target foods (Edelson et al., 2016). In a study comparing different types of prompts to eat, modelling was the approach that most successfully convinced toddlers to eat the target food (*n* = 60 children, aged 12–36 months; Edelson et al., 2016). However, when parents of CWDS were surveyed about modelling during feeding (*n* = 25 of 40 CWDS, aged 7–63 months), the average response was that the parents *slightly agreed* that they actively demonstrate healthy eating for the child (from a set of four questions; Melbye et al., 2011; Rogers et al., 2022). Parental modelling in this study was coded as ASP and noted by the coders in the respective video period's comment section; thus, the report of the direct success of modelling was limited. A total of 174 counts of modelling were recorded across both days of the study, and primarily recorded during food engagement—the video period where the child was eating the food product or had rejected the product and the parent was encouraging them to eat. Modelling was mostly accompanied by another ASP. For example, some parents would eat the product in front of their child, then say, ‘Your turn to try!’ and then wait for a response to their action and prompt.

The large amount of Neutral Talking recorded during the video coding may be attributed to parents, siblings, or others having discussions during the feeding session. The frequency of these Neutral Talking instances was coded as they are a part of the feeding environment and experience for the child eating, as these practices may keep children engaged with the feeding session and attentive to their environment. Previous findings from Fogel et al. (2019) have shown a relationship between the frequency of talking and eating speed.

Our second hypothesis was that no differences existed in the amount and type of feeding practices used on Day 2 versus Day 5. This hypothesis was incorrect since parents gave significantly more feeding practices earlier than later during the home‐use test. Parents and children may have become more familiar with the feeding study procedures by Day 5, which may explain why fewer practices were observed on this day. Parents may have also become more comfortable with feeding in front of the camera and felt less need to ‘perform’ as the study progressed. Another reason for this result may be due to the bidirectionality of child feeding (Fogel et al., 2018; Quah et al., 2018, 2019). Parents may adapt their feeding practices in response to the child. If their approach is successful, then the parent may keep performing the same practice; if their approach is unsuccessful, then a change in feeding practice may be observed.

In the present study, we also accounted for children directly fed by their parents. The children fed by their parents did not appear to have the gross and fine motor skills foods (e.g., poor coordination and weak pincer grasp) required to feed themselves for some or all of the textured snacks. Parents who fed their children used more ASP, CCP, Instruction and Positive Talking than the parents of children feeding themselves independently. This suggests that parents who are physically feeding their children are generally interacting more with the child, across the different types of feeding practices.

The parent–CWDS relationship during feeding was important to explore since there are no known studies that explore this relationship with observational methods (Nordstrøm et al., 2020). Perhaps the reason that observational studies are scarce in the context of feeding is due to recruitment (Surette et al., 2021). Recruiting a specialised population is a common challenge shared by previous studies with CWDS (Gisel et al., 1984a, 1984b; Spender et al., 1996), with obstacles such as geographical location and feasibility of logistics to travel to an on‐site evaluation location. This study was the first of its kind to explore the parent–CWDS relationship during feeding with observational measurements. Another strength of this study was the number of parent–CWDS dyads observed (*N* = 111). Of the few studies that explored parenting practices and parenting dimensions in CWDS, study populations have ranged from 10 to 35 mother–child dyads (Blacher et al., 2013; Gilmore & Cuskelly, 2012; Phillips et al., 2017). Our larger sample size provided the adequate statistical power needed to draw meaningful conclusions (Surette et al., 2021). Additionally, we recruited a hard‐to‐reach population nationwide through social media and by using the snowball method (Surette et al., 2021). The in‐home nature of the study meant that a larger and more widespread population could be recruited.

In addition to longitudinal assessment, future research could conduct an intervention using ASP (e.g., modelling and positive reinforcement) with a cohort of high food rejecting/challenging feeding CWDS. If such an intervention could increase food acceptance and intake with this cohort of CWDS, this would confirm the desirability of these practices for feeding CWDS.

As with all studies, this study experienced several limitations. First, this study was conducted with video data from a home‐use test where CWDS evaluated snack products, and thus, results may not generalise a typical family meal (with vegetables, novel foods; Moding & Fries, 2020). Next, future work should include questionnaires that measure parenting styles and parent behaviours/practices. Questions related to parental concerns about childhood obesity (O'Neill et al., 2005), parental stress level (Phillips et al., 2017), and choking concerns (Spender et al., 1996) should also be included in future work as these factors may influence parent behaviours/practices.

Also, exploring socioeconomic status and access to support for parents with CWDS may be important when understanding parent‐feeding practices (Marshall et al., 2015; Phillips et al., 2017). Differences in socioeconomic status may affect the access of support to services for CWDS (e.g., cost and quality of medical and therapeutical services, Caldwell & Krause, 2021).

The coding scheme was designed to count the number and type of feeding practices observed during a feeding session. For Neutral Talking, it was not possible to differentiate between child‐directed speech and background conversations. All counts of talking during the feeding session were counted since this practice was a part of the feeding environment. When an ‘Other’ prompt was observed, the coder was directed to describe the specific prompt in the comment section of the scheme; however, this was not consistently completed. As such, the interpretation and categorisation of these prompts into food‐related ‘Other’ prompts and non‐food‐related ‘Other’ prompts was slightly limited. We also may have missed parental modelling occurring behind the video recording device, potentially underestimating this behaviour.

## 5 CONCLUSION

This is the first study to explore parental practices used during feeding of young CWDS using observational methods, providing more context to CWDS feeding experiences. A coding scheme was developed to focus on feeding practices used by parents of CWDS during feeding of variously textured solid snack food products. We observed both the feeding practices used by parents and the CWDS response to those parent practices. We observed that autonomy‐supportive prompts were more likely to convince CWDS to eat the target food than coercive‐controlling prompts. Parents and feeding practitioners for CWDS should consider using more supportive feeding practices, such as modelling desired feeding behaviours, to encourage acceptance and successful intake in this population. Future research can build upon the knowledge gained from this study to confirm longitudinal associations of parent practices with child behaviours during feeding.

## AUTHOR CONTRIBUTIONS

Victoria A. Surette performed the research. Victoria A. Surette and Carolyn F. Ross designed the research study. Sarah Smith‐Simpson, Lisa R. Fries and Ciarán G. Forde contributed essential guidance and input for the research study. Victoria A. Surette and Carolyn F. Ross analysed the data. Victoria A. Surette wrote the paper. Carolyn F. Ross, Sarah Smith‐Simpson, Lisa R. Fries and Ciarán G. Forde edited the paper.

## CONFLICT OF INTEREST STATEMENT

The authors declare no conflict of interest.

## Data Availability

The data that support the findings of this study are available from the corresponding author upon reasonable request.
